# Optimizing the Ratio of Metallic and Single-Atom Co in CoNC via Annealing Temperature Modulation for Enhanced Bifunctional Oxygen Evolution Reaction/Oxygen Reduction Reaction Activity

**DOI:** 10.3390/molecules29235721

**Published:** 2024-12-04

**Authors:** Hengxu Cheng, Haojie Sun, Meizhen Dai, Yucai Li, Jian Wang, Shiwei Song, Dong Zhang, Depeng Zhao

**Affiliations:** School of New Energy, Shenyang Institute of Engineering, Shenyang 110136, China

**Keywords:** CoNC, pyrolysis temperature, oxygen reduction reaction, oxygen evolution reaction, rechargeable Zn-air battery

## Abstract

Developing low-cost, efficient alternatives to catalysts for bifunctional oxygen electrode catalysis in the oxygen reduction reaction (ORR) and oxygen evolution reaction (OER) is critical for advancing the practical applications of alkaline fuel cells. In this study, Co particles and single atoms co-loaded on nitrogen-doped carbon (CoNC) were synthesized via pyrolysis of a C_3_N_4_ and cobalt nitrate mixture at varying temperatures (900, 950, and 1000 °C). The pyrolysis temperature and precursor ratios were found to significantly influence the ORR/OER performance of the resulting catalysts. The optimized CoNC-950 catalyst demonstrated exceptional ORR (E_1/2_ = 0.85 V) and OER (E_j10_ = 320 mV) activities, surpassing commercial Pt/C + RuO_2_-based devices when used in a rechargeable zinc–air battery. This work presents an effective strategy for designing high-performance non-precious metal bifunctional electrocatalysts for alkaline environments.

## 1. Introduction

Zinc–air batteries (ZABs) are regarded as one of the most promising energy storage devices due to their low cost, sustainability, and high energy density [1,2,3]. The oxygen evolution reaction (OER) and oxygen reduction reaction (ORR) are critical to the charge/discharge processes of ZABs [4,5]. While benchmark catalysts (e.g., IrO_2_, RuO_2_, Pt/C) demonstrate high activity in accelerating these lower reaction kinetics, their commercial use is limited by high costs and performance degradation [6,7,8]. More importantly, these catalysts are inefficient at simultaneously facilitating both reactions [9,10,11]. Therefore, the development of high-performance catalysts capable of efficiently driving both reactions within a single cathode is a challenging yet highly valuable scientific and economic pursuit.

To optimize the catalytic performance, the rational design of electrocatalysts capable of simultaneously driving both reactions has become a major research focus. In the case of ORR, substantial evidence indicates that metal–nitrogen–carbon (MNC, M=Fe, Co) structures can lower OOH and OH adsorption energies, thereby improving ORR activity [12,13,14]. For example, Tipaporn et al. synthesized MNx structures with various metals (Cr, Mn, Fe, Co, Ni, Ru) to explore stability and catalytic activity, finding them thermodynamically unstable in acidic ORR conditions [15]. Among various M-N-C structures, theoretical calculations suggest that CoNC shows the most promise as a bifunctional catalyst [16,17,18]. Furthermore, metal nanoparticles have been identified as highly active OER sites, offering enhanced activity and stability when combined with carbon compared to individual metal elements.

The combination of CoNC structures and metal nanoparticles within a single bifunctional composite catalyst is expected to deliver superior ORR and OER performance [19,20,21]. In this study, a series of bifunctional oxygen electrocatalysts, consisting of nitrogen-doped carbon (NC)-encapsulated Co particles, were synthesized through a simple pyrolysis. The incorporation of Co promoted the formation of M-N_x_ active sites, enhanced ORR activity, and facilitated the creation of surface reconstruction layers during OER. The bifunctional activity of the CoNC catalysts was optimized by adjusting the annealing temperature. Consequently, CoNC demonstrated excellent ORR performance, with a half-wave potential (E_1/2_) of 0.85 V, and enhanced OER activity, exhibiting an overpotential of 320 mV (10 mA cm^−2^) and a ΔE value of 0.70 V. Furthermore, the ZABs with CoNC-950 showed higher power density (181 mW cm^−2^) and superior long-term stability compared to devices utilizing precious metal catalysts (Pt/C + RuO_2_). The study presents a feasible strategy for synthesizing bimetallic nitrogen–carbon catalysts.

## 2. Results and Discussion

**Figure 1:**
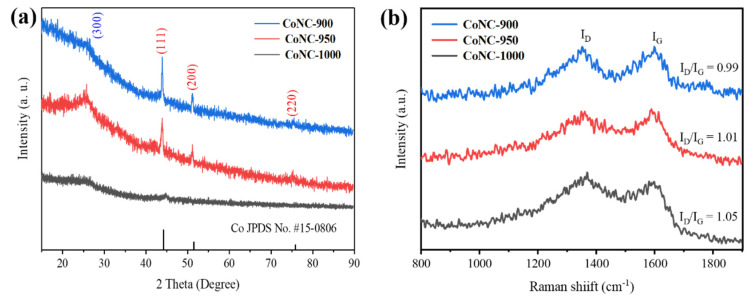
XRD patterns of (**a**) CoNC-900, CoNC-950, and CoNC-1000. (**b**) Raman spectra of CoNC-900, CoNC-950, and CoNC-1000.

X-ray diffraction (XRD) patterns reveal four distinct diffraction peaks for CoNC (Figure 1a). The three prominent peaks at approximately 44.1°, 51.2°, and 75.6° correspond to metallic Co (JCPDS No. 15-0806) [16], indicating high crystallinity of cobalt in the synthesized sample. A peak at 26.4° is attributed to the (300) plane of disordered graphite, suggesting the presence of a graphite carbon phase in the sample [22]. Notably, as the pyrolysis temperature increases, the intensity of metallic Co peaks significantly rises and the peaks become sharper. This indicates an increase in the grain size of metallic Co and improved crystallinity at higher temperatures, which facilitates cobalt crystal growth and the formation of a more ordered structure. However, higher pyrolysis temperatures also lead to some carbon volatilization, reducing the carbon content and potentially affecting the sample’s electrical conductivity and structural stability [23]. Therefore, controlling the pyrolysis temperature allows for the regulation of both the crystallinity of metallic Co and the composition and structure of the carbon phase, thereby optimizing the material’s electrocatalytic performance.

The transformation of carbon in CoNC is confirmed by Raman spectroscopy (Figure 1b), which shows peaks at 1350 cm^−1^ (D band) and 1590 cm^−1^ (G band). The D band indicates a significant presence of disordered or defective carbon, while the G band represents regions with higher graphitization [24,25]. As the annealing temperature increases, the intensity ratio of the D band to the G band rises from 0.99 to 1.05, signifying an increase in structural defects in the carbon. This rise in defects is likely due to the rearrangement of carbon atoms and the loss of some carbon phases at elevated temperatures. The graphitic structure of carbon enhances the sample’s electrical conductivity, facilitating electron transfer during electrocatalytic reactions. Additionally, the structural defects signaled by the D band not only impact the conductivity but also provide additional active sites, thereby contributing to the overall catalytic activity. Consequently, during the preparation of CoNC catalysts, optimizing the annealing temperature is crucial for balancing the degree of graphitization and defect content, thereby achieving an ideal combination of conductivity and catalytic performance. An appropriate level of defects ensures both high electrical conductivity and sufficient active sites, resulting in excellent performance.

**Figure 2:**
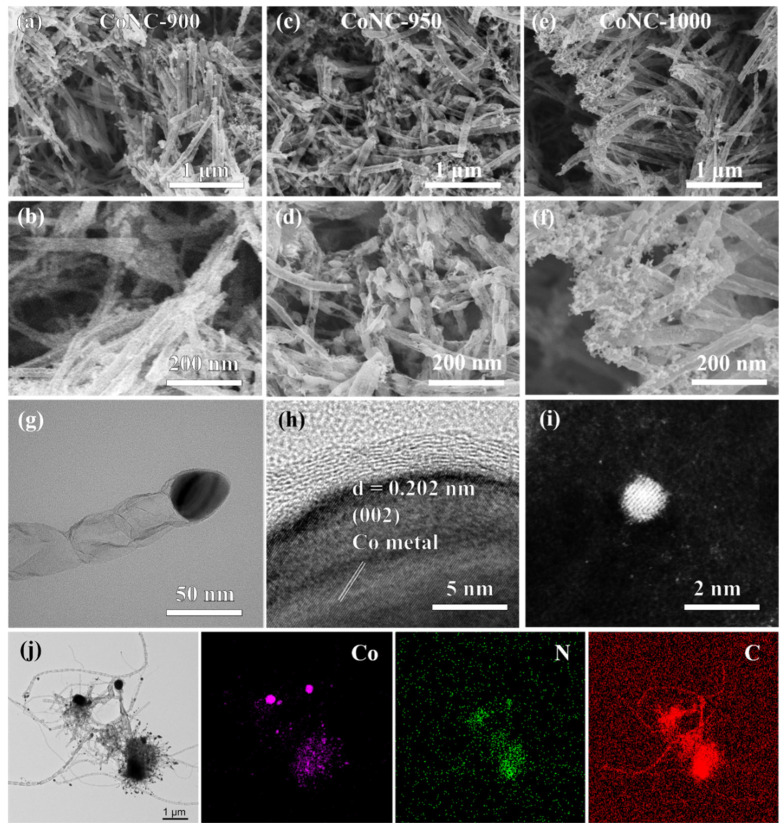
SEM images of (**a**,**b**) CoNC-900, (**c**,**d**) CoNC-950, and (**e**,**f**) CoNC-1000, (**g**) TEM images of CoNC-950, (**h**) HRTEM image of CoNC-950, (**i**) HAADF-STEM image of CoNC-950. (**j**) Energy-dispersive X-ray spectroscopy mapping imageries of CoNC-950.

A series of CoNC samples were synthesized using a straightforward annealing method, which involved coating CoNi metal particles with NC. SEM images (Figure 2) reveal that all samples exhibit nanowire and metal particle morphologies. Following annealing, the carbon matrix predominantly forms a nanowire structure, with the metal particles uniformly distributed within these carbon tubes. Specifically, at an annealing temperature of 900 °C, the metal particles are relatively small and are evenly coated inside the carbon tubes, creating a compact core–shell structure. This tight coating enhances the material’s stability while providing good electrical conductivity and active sites for electrocatalytic reactions. As the annealing temperature increases, the size of the metal particles grows significantly, indicating that metal grains undergo growth and aggregation at higher temperatures. At an annealing temperature of 1000 °C, some metal particles detach from the carbon tube shell due to intensified thermal motion, resulting in exposed metal particles on the surface of the carbon nanotubes.

This morphological change not only affects the material’s physical properties but also significantly impacts its catalytic performance. Different morphologies have a pronounced effect on catalytic activity. Uniformly distributed metal particles offer more active sites, facilitating the reaction, while the nanowire structure enhances conductivity and mass transfer efficiency. However, as particle size increases or particles detach from the carbon tubes, the specific surface area decreases, and the number of sites is reduced, potentially leading to a decline in catalytic performance. Therefore, precise control over the annealing temperature allows for optimal tuning of the nanowire and metal particle morphology, thereby enhancing the material’s electrocatalytic activity. The transmission electron microscopy (TEM) images (Figure 2g) provide a detailed view of the morphology, revealing that metallic cobalt (Co) particles are tightly encapsulated within carbon nanotubes. It is clearly observable that these cobalt particles are uniformly embedded within the carbon nanotube structure, forming a unique nano-composite material with distinct phase characteristics.

A precise analysis of the lattice spacing further confirms the presence and crystalline structure of cobalt (Figure 2f). Specifically, the measured lattice spacing of 0.202 nm corresponds to the (002) crystal plane of metallic cobalt. This result not only verifies that the observed particles are indeed metallic cobalt but also reveals their ordered arrangement within the carbon nanotubes, offering critical microscopic evidence for understanding the structure and potential electrocatalytic performance of the material. It can be observed from Figure 2i that the prepared CoNC features a structure with the coexistence of single atoms and clusters. The energy-dispersive X-ray spectroscopy mapping imageries of the prepared sample are shown in Figure 2j. From the image, it can be observed that Co, N, and C elements are uniformly distributed on the surface of the carbon nanotubes. The large-scale accumulation of Co elements on the surface of the metal particles confirms that the metal particles are composed of Co metal.

**Figure 3:**
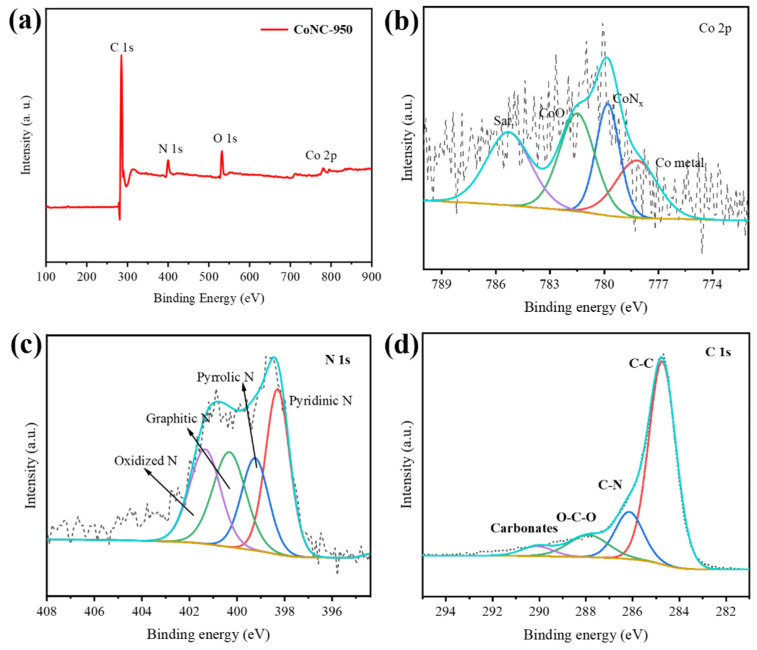
XPS spectra of CoNC-950 samples (**a**) survey spectrum, (**b**) Co 2p, (**c**) N 1s, and (**d**) C 1s.

The X-ray photoelectron spectroscopy (XPS) full spectrum of CoNC-950 (Figure 3a) displays characteristic peaks at C1s, N1s, and Co2p, confirming the successful incorporation of C, N, and Co elements into the carbon-based material. These results also verify the presence of metallic cobalt and nitrogen within the carbon matrix. The Co2p spectrum (Figure 3b) reveals various chemical states of cobalt, with peaks corresponding to metallic Co, CoN_x_, and CoO, with a typical satellite peak at 785.4 eV. These features indicate the existence of Co-N species, which are believed to serve as active sites in catalytic reactions [26,27]. Furthermore, the oxidized Co is likely due to exposure to the atmosphere, consistent with the material’s surface sensitivity. The N1s spectrum (Figure 3c) indicates several nitrogen species, including pyridinic N, pyrrolic N, graphitic N, and oxidized N [28,29,30]. Pyridinic and pyrrolic nitrogen are generally considered more catalytically active, while graphitic nitrogen enhances the material’s conductivity [15,31]. The presence of oxidized nitrogen suggests some surface oxidation. Analysis of the N1s spectrum clearly demonstrates the doping of nitrogen into the matrix, forming N-C structures [32,33]. The high-resolution C1s spectrum (Figure 3d) further reveals the bonding states within the carbon material, with peaks corresponding to C–C, C–N, O-C-O bonds, and carbonates (290.5 eV). The presence of C–C bonds suggests that the carbon in the sample primarily exists as amorphous carbon, further confirming the conversion of g-C_3_N_4_; into amorphous carbon during thermal treatment [34,35]. This result aligns with the XRD and Raman spectroscopy findings, which show the degree of graphitization and structural defects in the sample. These analyses indicate that the CoNC-950 material prepared by the two-step annealing method successfully incorporates cobalt and nitrogen, while also exhibiting good conductivity and a well-distributed network of active sites, making it a promising candidate for electrocatalytic applications. The BET characterization of CoNC-950 shows a specific surface area of 241 cm^2^/g, indicating its high catalytic activity potential. The nitrogen adsorption–desorption isotherm analysis reveals a pore size of approximately 3 nm, which facilitates the effective transport of reactants and products, ensuring accessibility to the catalytic sites. The combination of high specific surface area and suitable pore size demonstrates CoNC-950’s excellent catalytic performance in energy-related applications such as electrocatalysis, confirming its effectiveness as a catalyst.

**Figure 4:**
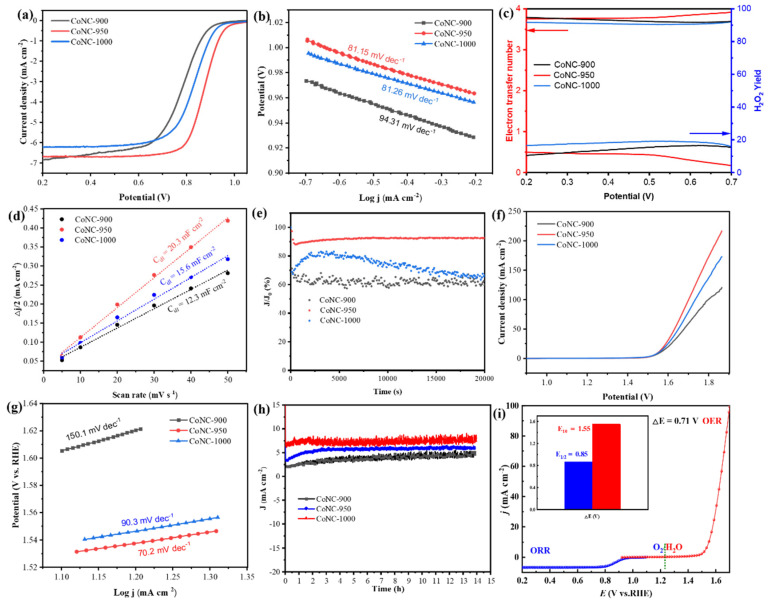
ORR performances of the CoNC-900, CoNC-950 and CoNC-1000. (**a**) LSV curves in O_2_-saturated 0.1 M KOH electrolyte and (**b**) corresponding Tafel slopes. (**c**) Electron transfer number and H_2_O_2_ yield. (**d**) Cdl values. (**e**) i-t curves of ORR. OER performances of the above catalysts. (**f**) Lsv curves and (**g**) corresponding Tafel slopes. (**h**) i-t curves of OER. (**i**) ΔE of ORR and OER.

The linear sweep voltammetry (LSV) curves for CoNC-950 demonstrate that the optimized CoNC-950 catalyst exhibits outstanding catalytic performance at 0.23 mg cm^−2^. The optimal catalyst loading ensures full utilization of active materials while achieving a balance between performance and cost. As shown in Figure 4a, CoNC-950 shows excellent catalytic activity in ORR, with an onset potential (*E_onset_*) of 1.04 V, significantly higher than CoNC-900 (*E_onset_* of 0.95 V) and CoNC-1000 (*E_onset_* of 0.99 V). This indicates that the catalytic sites in the CoNC-950 sample, synthesized at 950 °C, are optimally activated. Furthermore, CoNC-950 achieves an E_1/2_ of 0.85 V, superior to CoNC-900’s 0.80 V and CoNC-1000’s 0.81 V, further confirming its excellent ORR activity under alkaline conditions. Additional analysis reveals that CoNC-950 exhibits rapid kinetics, with a Tafel slope of 81.15 mV dec^−1^, indicating efficient reaction rates at lower overpotentials. In comparison, CoNC-900 has a Tafel slope of 94.31 mV dec^−1^, and CoNC-1000 has a slope of 81.26 mV dec^−1^, showing that annealing temperature influences the microstructure and electrochemical performance. The appropriate annealing temperature not only optimizes the pore structure and distribution of active sites in CoNC but also enhances the interaction between nitrogen-doped carbon materials’ active centers and oxygen molecules in the electrolyte, thus significantly improving catalytic activity.

To further confirm the electron transfer number and hydrogen peroxide (H_2_O_2_) yield of the prepared catalysts, detailed electrochemical tests were performed (RRDE, Figure 4c). The results show that the electron transfer number of CoNC-950 is close to 4, indicating that ORR predominantly follows a highly efficient four-electron pathway. Additionally, the calculated H_2_O_2_ yield in 0.20 to 0.70 V is approximately 10%, the lowest among the three samples, suggesting that CoNC-950 effectively suppresses H_2_O_2_ formation from incomplete reduction. In contrast, the other samples exhibit higher H_2_O_2_ yields, highlighting CoNC-950’s advantage in minimizing byproduct generation. The low H_2_O_2_ yield indicates better selectivity and stability, allowing the catalyst to maintain high efficiency over a broad potential range. Overall, these results demonstrate CoNC-950’s superior performance for ORR, with both rapid electron transfer and minimal byproduct formation, underscoring its potential for efficient catalytic applications.

The electrochemical surface area (ECSA) was determined by measuring the double-layer capacitance (C_dl_) from cyclic voltammetry (CV) curves (Figure S1) in the non-faradaic region. CoNC-950 exhibited a C_dl_ value of 20.3 mF cm^−2^, higher than CoNC-900 (12.3 mF cm^−2^) and CoNC-1000 (15.6 mF cm^−2^), indicating a larger ECSA for CoNC-950 due to the exposure of more active sites. Long-term stability tests at a constant potential of 0.85 V that CoNC-950 retained approximately 98.5% of its initial current density after 20,000 s of continuous operation, demonstrating excellent electrochemical stability. In comparison, CoNC-900 and CoNC-1000 retained only 87.2% and 89.7% of their initial current densities, respectively, under the same conditions. This finding indicates that CoNC-950 not only delivers superior catalytic performance initially but also maintains excellent stability and resistance to degradation over time. The lower rate of current decay suggests that CoNC-950 can extend its service life in practical applications and reduce performance losses due to catalyst deactivation, making it a highly promising catalyst. For the ORR, the presence of Co particles promotes the saturation of single-atom CoNx structures on the carbon substrate, effectively increasing the content of the active sites CoNx for the ORR. This saturation state not only enhances the activity of the catalyst but also improves its adsorption capacity for reactants, further increasing the reaction efficiency. In the OER, a large number of CoNx structures undergo reconstruction to form stable Co–O bonds, which are crucial for the OER process. Simultaneously, the Co metal particles also participate in the reconstruction, creating new active sites. The synergistic effect of these two types of active sites allows CoNx and Co metal particles to work together, enhancing the catalytic performance of the OER and facilitating more efficient electrochemical reactions. This mutually reinforcing mechanism not only optimizes the performance of the catalyst but also provides new insights for designing more efficient catalysts.

We conducted a detailed evaluation of OER electrocatalytic activity. Figure 4f presents the polarization curves. The results demonstrate that CoNC-950 exhibits an overpotential of 320 mV, significantly lower than that of CoNC-900 (370 mV) and CoNC-1000 (350 mV), underscoring CoNC-950’s superior performance in enhancing current density. Additionally, the Tafel plot in Figure 4g confirms the excellent OER performance of CoNC-950, showing a Tafel slope of 70.2 mV dec^−1^, which is much lower than that of CoNC-900 (150.1 mV dec^−1^) and CoNC-1000 (90.3 mV dec^−1^), indicating faster reaction kinetics. These findings highlight the advantages of CoNC-950 in terms of catalytic efficiency and reaction kinetics. Electrochemical impedance spectroscopy (EIS) (Figure S1) confirms that among the three catalysts, CoNC-950 has the smallest arc, indicating the lowest charge transfer resistance and the best conductivity [36].

Long-term durability is a critical factor in evaluating the practical application. As shown in Figure 4h, CoNC-950 exhibits no significant current decay after 10 h of testing in 1.0 M KOH, demonstrating excellent stability. The bifunctional catalytic activity was assessed by comparing the potential difference (ΔE) between OER potential at *E_j=10_* and ORRE_1/2_. A smaller ΔE typically indicates superior bifunctional electrocatalytic performance. As illustrated in Figure 4i, CoNC-950 achieves a ΔE of 0.70 V, which is significantly lower than that of CoNC-900 (0.80 V) and CoNC-1000 (0.77 V). The results show a clearly outstanding bifunctional performance of CoNC-950. In the XRD patterns after testing, the diffraction peaks associated with metallic Co in the prepared sample became weaker, indicating partial degradation of the Co metal during the testing process. The XPS results showed no significant changes.

**Figure 5:**
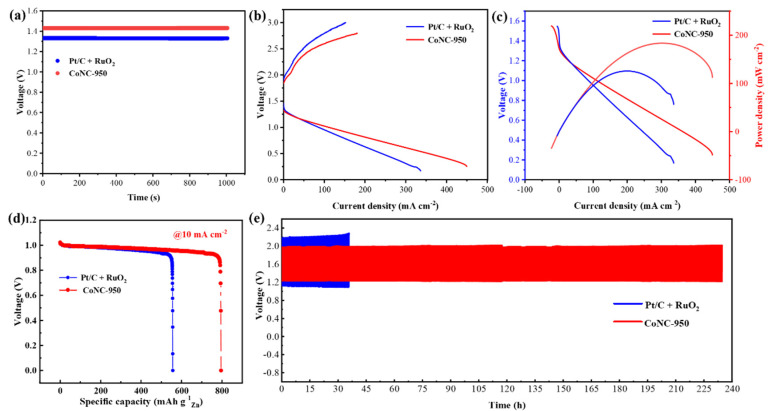
(**a**) Open-circuit voltages of CoNC-950 and Pt/C + RuO_2_-based zinc–air batteries. (**b**) Charging-discharging polarization plots of the batteries. (**c**) Discharge polarization curves and the corresponding power density curves of the above two samples. (**d**) Galvanostatic discharge curves at a current density of 10 mA cm^−2^. The specific capacity was calculated based on the mass loss of consumed Zn. (**e**) Galvanostatic charging/discharging cycling curves at 10 mA cm^−2^.

To validate the potential application of the CoNC-950, a ZAB was assembled using CoNC-950 as the air cathode, and a zinc plate as the anode. Pt/C + RuO_2_ (1:1 mass ratio) was used as a reference alongside the CoNC-950 catalyst. The battery based on CoNC-950 (Figure 5a) exhibited a high open-circuit voltage of 1.43 V, surpassing the Pt/C + RuO_2_ catalyst, which demonstrated 1.32 V. Discharge and charge polarization curves were recorded, revealing that the CoNC-950-based ZABs had a smaller voltage gap, indicating excellent rechargeability (Figure 5b), the battery (Figure 5c) based on CoNC-950 exhibited a maximum peak power density of 181 mW cm^−2^, significantly higher than the Pt/C + RuO_2_-based battery, which achieved only 122 mW cm^−2^. Figure 5d shows that the specific capacity of the CoNC-950-based battery (10 mA cm^−2^) was 796.9 mAh g^−1^, whereas the Pt/C + RuO_2_-based battery only reached 559.1 mAh g^−1^ under the same conditions. Furthermore, CoNC-950 exhibited excellent long-term durability, with almost no voltage decay over 235 h, demonstrating superior longevity compared to Pt/C + RuO_2_-based battery (Figure 5e), along with high and stable cycling efficiency. The failure modes of CoNC containing Co particles and single-atom sites in zinc–air batteries during cycling include catalyst deactivation, structural changes, electrolyte corrosion, zinc deposition and corrosion, interface failure, and changes in zinc anode morphology, all of which lead to a gradual decline in catalytic activity and overall battery performance. The zinc–air battery assembled with CoNC-950 showed superior performance in terms of peak power density, specific capacity, and cycling stability, outperforming benchmark batteries. The outstanding bifunctional ORR and OER performance in rechargeable ZABs is attributed to the synergistic effect between the single-atom catalysts and Co particles, obtained at different synthesis temperatures. In conclusion, these results strongly support the superiority of CoNC-950 as a promising sample for zinc–air batteries. Our catalyst outperforms various recent non-precious metal bifunctional oxygen electrode catalysts in terms of ORR and OER potential differences, and demonstrates superior maximum power density in rechargeable zinc–air batteries under prolonged cycling conditions, as shown in Table S1.

## 3. Experimental

Melamine (99.99%) and cobalt nitrate hexahydrate (Co(NO_3_)_2_·6H_2_O, ≥99.00%) were purchased from Tianjin Damao Chemical Reagent Co., Ltd. (Tianjin, China). All reagents were used as purchased without further purification.

### 3.1. Synthesis of CoNC

Melamine was heated to 450 °C for 2 h. Subsequently, 1 g of Co(NO_3_)_2_·6H_2_O was ground together with 1 g of pre-synthesized g-C_3_N_4_. The mixture was heated to 900 °C, 950 °C, and 1000 °C at 3 °C/min under an argon atmosphere, and held at each temperature for 2 h. The annealed samples were then soaked in 1.0 M H_2_SO_4_ for 12 h and collected by centrifugation. Finally, the samples were air-dried for 12 h.

### 3.2. Materials Characterization

Scanning electron microscopy (SEM) was conducted using a Hitachi S-4800 (Tokyo, Japan) microscope operating at 10 kV. X-ray diffraction (XRD) patterns were recorded on a D8 Advance powder diffractometer (Bremen, Germany). Raman spectroscopy measurements were carried out using a GX-PT-1500 spectrometer (Bremen, Germany) with a 532 nm laser (approximately 1 mW power). X-ray photoelectron spectroscopy (XPS) analysis was performed on an ESCALAB 250Xi system (Thermo Fisher Scientific, Waltham, MA, USA) with a power setting of 120 W.

### 3.3. Electrochemical Measurements

Electrochemical measurements were conducted at room temperature using a CH Instruments CHI760E electrochemical workstation (Chenghua, Shanghai, China) with a three-electrode setup. A rotating ring-disk electrode (RRDE) modified with CoNC (Pine American, Hong Kong, China), which features a carbon disk with a surface area of 0.2475 cm^2^ and a platinum ring with a surface area of 0.1866 cm^2^, served as the working electrode. A Hg/HgO (3.5 M KCl) electrode and a platinum electrode (Yueci, Shanghai, China) were used as the reference and counter electrodes, respectively. To prepare the working electrode, 10 mg of CoNC was ultrasonicated in a mixture of 1.25 mL ethanol and 30 μL of 5% Nafion for 30 min to create a catalyst ink. The resulting suspension was then deposited onto a polished glassy carbon electrode and allowed to dry.

For OER, electrochemical studies were conducted at room temperature using cyclic voltammetry (CV) and linear sweep voltammetry (LSV) techniques. The oxygen evolution reaction (OER) measurements were performed in an oxygen-saturated 0.1 M KOH solution. Linear sweep voltammetry (LSV) involves applying a linearly varying voltage to the electrode and recording the current as it changes with the voltage. In this study, the prepared sample is used as the working electrode, and a Hg/HgO electrode is employed as the reference electrode, with calibrations conducted before and after the experiments. All voltages of the curves are calculated with respect to the reversible hydrogen electrode (RHE), and the potential calculation formula for the RHE is as follows:

*E_RHE_* = *E_Hg/HgO_* + 0.098 V+ 0.059 V × pH (1)

where *E_RHE_* is the potential of the reversible hydrogen electrode (RHE), and *E_Hg/HgO_* represents the experimental measured potential relative to the Hg/HgO reference electrode. The CoNC catalyst was applied to the working electrode at a loading of 0.2 mg cm^−2^ in this solution. CV, LSV, and chronoamperometric measurements were carried out at a scan rate of 10 mV s^−1^. The oxygen evolution reaction (OER) performance of the catalyst was assessed in a 1 M KOH solution at a scan rate of 5 mV s^−1^, with iR correction applied, and a total catalyst loading of 0.36 mg cm^−2^.

For ORR, CV polarization curves were recorded from 0.2 to 1.0 V vs. Hg/HgO at 50 mV s^−1^ in a N_2_/O_2_-saturated 0.1 M KOH. Linear sweep voltammetry (LSV) was obtained in the same potential range with various rotation speeds (100, 400, 900, 1600 and 2500 rpm) at 10 mV s^−1^ and the ring was constantly polarized at 1.3 V. The potentials were estimated according to the following equation:

(2) $$E_{RHE}=E_{0}+E_{Hg/HgO}+0.059\times pH-I_{d}R_{s}$$

where *E*_0_ represents the measured potential and *E_Hg/HgO_* is 0.098 V, *I_d_* denotes the disk current and *R_s_* is the resistance of the electrolyte.

The electron transfer number (*n*) was calculated by the diffusion limiting current density (*j_L_*) at different rotating speed (*ω*) according to Koutecky–Levich plots according to the following equation:

(3) $$\frac{1}{j}=\frac{1}{j_{L}}+\frac{1}{j_{K}}=\frac{1}{B\omega^{\frac{1}{2}}}+\frac{1}{j_{K}}=\frac{1}{(0.2nFC_{0}{D_{0}}^{\frac{2}{3}}\nu^{-\frac{1}{6}})\omega^{\frac{1}{2}}}+\frac{1}{j_{K}}$$

where *F* and *ν* are the Faraday constant (96,485 C mol^−1^) and the kinematic viscosity of the electrolyte (0.01 cm^2^ s^−1^); *C*_0_ and *D*_0_ are the bulk concentration (1.2 × 10^−6^ mol cm^−3^) and diffusion coefficient (1.9 × 10^−5^ cm^2^ s^−1^) of O_2_ in 0.1 M KOH, respectively.

The value of *n* was also determined by calculating the H_2_O_2_ yield derived from the ring current. The H_2_O_2_ yield and *n* were calculated by the following equations:

(4) $$\%H_{2}O_{2}=200\frac{\frac{i_{r}}{N}}{i_{d}+\frac{i_{r}}{N}}=\;200\frac{i_{r}}{Ni_{d}+i_{r}}$$

(5) $$n=\;4\frac{i_{d}}{i_{d}+\frac{i_{r}}{N}}=\;4\frac{Ni_{d}}{Ni_{d}+i_{r}}$$

where *I_r_* and *N* are the ring current and the collection efficiency of the Pt ring (0.424).

For OER tests, CV experiments were conducted to reach a stable state within an O_2_-saturated 1 M aqueous KOH solution with a scan rate of 50 mV s^−1^. The LSV curves were recorded at a scan rate of 10 mV s^−1^ and further treated with 95% iR correction. Tafel slopes were calculated from the corresponding polarization curves at a low current density range by the following equation:

(6) η=blog⁡j+a

where *η*, *j* and *b* denote the overpotential, current density and Tafel slope, respectively.

### 3.4. Zinc–Air Battery

A 10 mg CoNC-950 catalyst ink was prepared by dispersing the catalyst in a mixture of 1.25 mL ethanol and 30 μL of 5% Nafion. Zinc–air batteries were tested using a laboratory-assembled electrochemical cell. For the primary battery setup, a carbon paper electrode (3.14 cm^2^) loaded with 10 mg of CoNC-950 served as the cathode, with a zinc sheet as the anode and an electrolyte composed of 6 M KOH and 0.2 M zinc acetate. All zinc–air batteries were evaluated under ambient conditions. Constant current charge–discharge cycling tests were performed at room temperature using a LAND CT2001 A battery system (Landian, Wuhan, China).

## 4. Conclusions

We successfully synthesized a highly efficient CoNC-950 catalyst for both ORR and OER through a straightforward pyrolysis method. C_3_N_4_ was used as a hard template, providing both nitrogen and carbon, while cobalt nitrate served as the cobalt source. The composition of the catalyst and the pyrolysis temperature were critical in achieving its bifunctional activity. The results show that CoNC-950 exhibits excellent performance, with an *E_1/2_* of 0.85 V for ORR and an overpotential of 320 mV for OER at 10 mA cm^−2^. When used as the air electrode in a zinc–air battery, CoNC-950 achieved a peak power density of 181 mW cm^−2^. Additionally, no significant loss of activity was observed after 235 h of continuous charge–discharge cycling. This study offers valuable insights for developing highly efficient oxygen electrocatalysts for energy storage.

## Data Availability

Data will be made available on request.

## Supplementary Materials

The following supporting information can be downloaded at: https://www.mdpi.com/article/10.3390/molecules29235721/s1, Figures S1–S5: Figure S1: (a) N_2_ adsorption-desorption isotherms and (b) pore size distribution of CoNC-950, Figure S2: The curves of (a) CoNC-900, (b) CoNC-950, and (c) CoNC-1000, Figure S3: EIS plots of CoNC-900, CoNC-950, and CoNC-1000, Figure S4: XRD of CoNC-950 samples after test, Figure S5: XPS spectra of CoNC-950 samples after test (a) survey spectrum, (b) Co 2p, (c) N 1s, and (d) C 1s; Table S1: These references [37,38,39,40,41,42,43,44] are cited in supplementary material.
