# Supplementary material for: Optimizing the Ratio of Metallic and Single-Atom Co in CoNC via Annealing Temperature Modulation for Enhanced Bifunctional Oxygen Evolution Reaction/Oxygen Reduction Reaction Activity
Source: Molecules. 2024 Dec 4;29(23):5721. doi: 10.3390/molecules29235721 (PMC11643407; doi:10.3390/molecules29235721)
Supplement: Supplementary file 1 [file molecules-29-05721-s001.zip › molecules-3279099-supplementary.pdf]

Supporting Information

Optimizing the Ratio of Metallic and Single-Atom Co in CoNC via Annealing Temperature Modulation for Enhanced Bifunctional Oxygen Evolution Reaction/Oxygen Reduction Reaction Activity

Figures and Tables

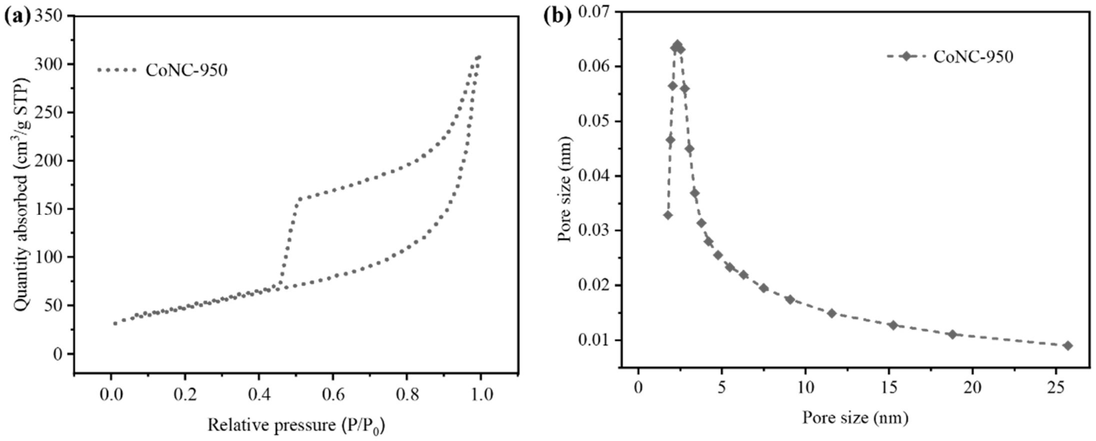

Figure S1. (a) N_2 adsorption-desorption isotherms and (b) pore size distribution of CoNC-950.

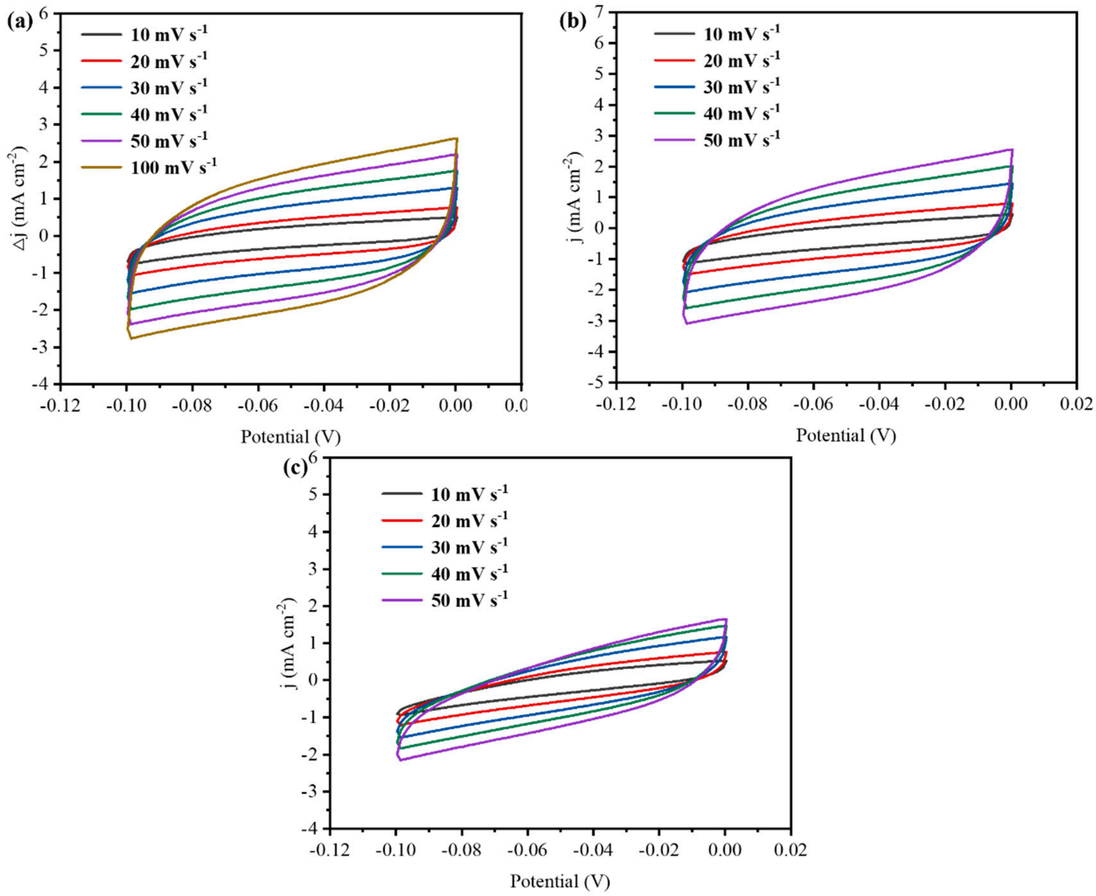

Figure S2. The curves of (a) CoNC-900, (b) CoNC-950, and (c) CoNC-1000.

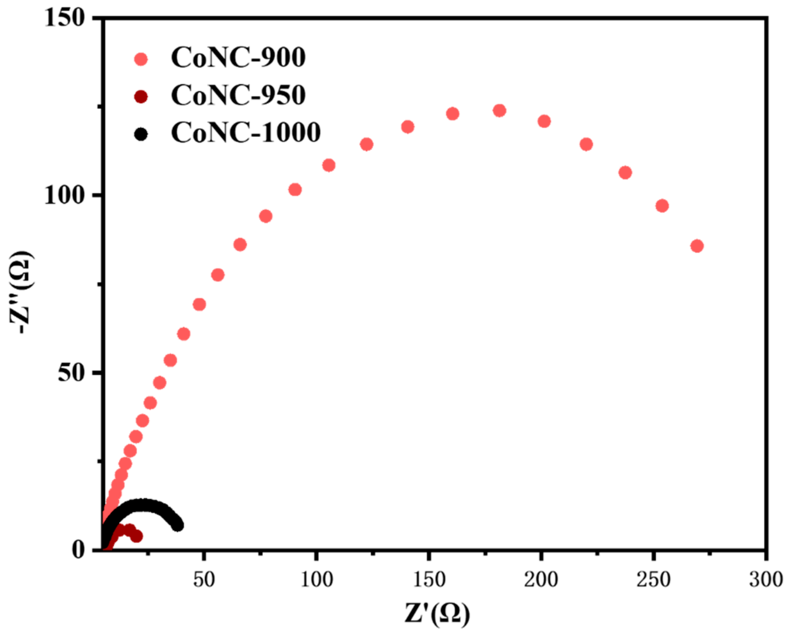

Figure S3. EIS plots of CoNC-900, CoNC-950, and CoNC-1000.

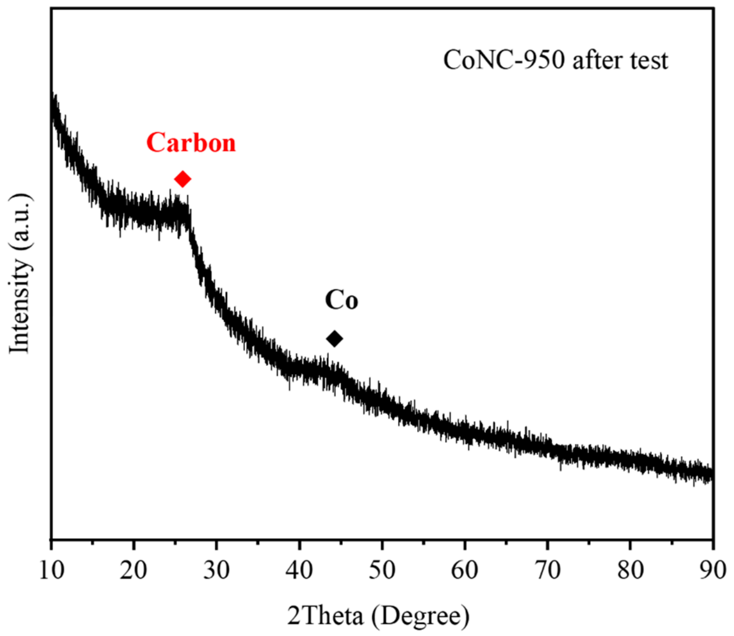

Figure S4. XPS spectra of CoNC-950 samples after test (a) survey spectrum, (b) Co 2p, (c) N 1s, and (d) C 1s.

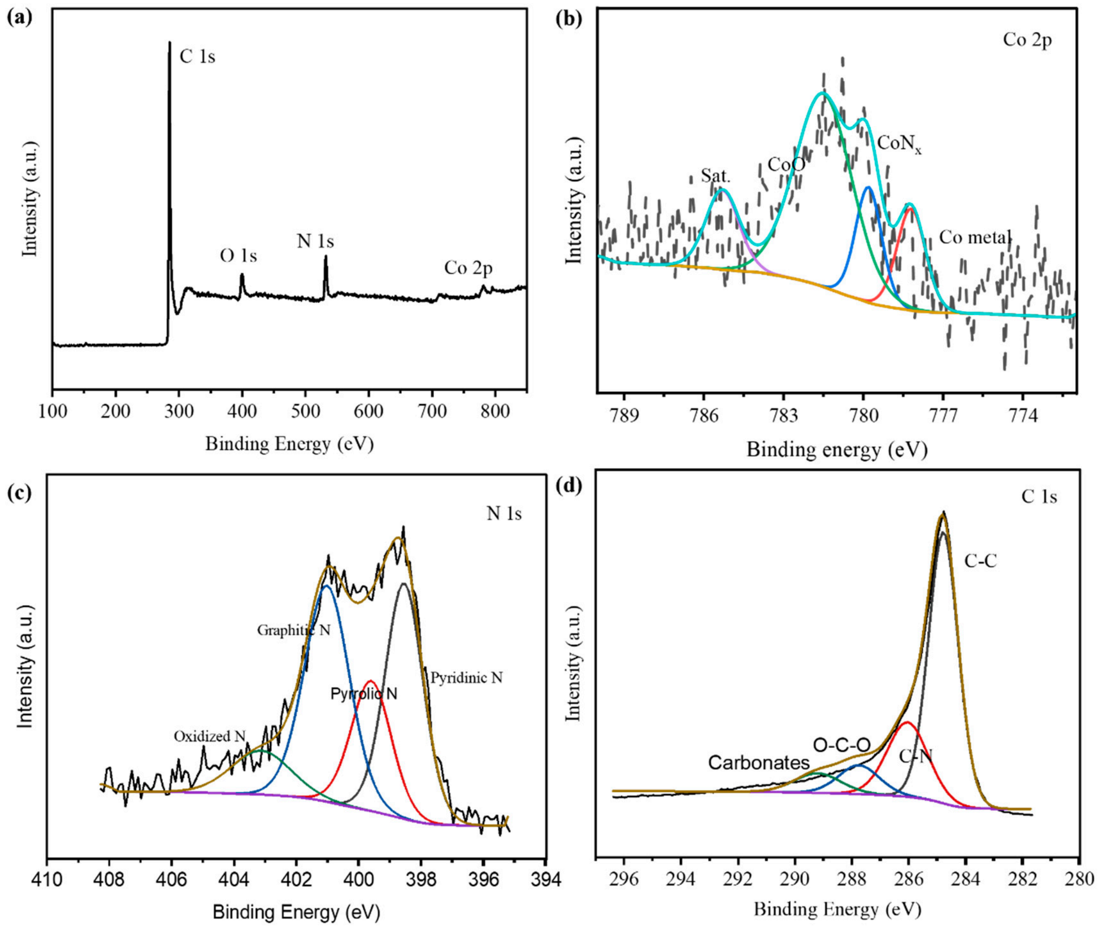

Figure S5. XPS spectra of CoNC-950 samples after test (a) survey spectrum, (b) Co 2p, (c) N 1s, and (d) C 1s.

Table S1. Comparison of the ORR, OER and Zn-air battery performance based on CoNC-950 and another related non-precious metal catalysts in literature

**Catalysts**	**$E_{1/2}$**	**η @ 10 mA cm^{-2}**	**△E**	**Power density (mW cm^{-2})**	**Ref.**
Co@H-NCNT	0.85 V	0.35 V	0.73 V	207.0	1
Fe-Se/NC	0.92 V	0.39 V	0.69 V	135.0	2
Co-N-CTS	0.84 V	0.29 V	0.68 V	140.0	3
CoFe-NCNFs	0.85 V	0.32 V	0.70 V	116.1	4
O-Co-N/C	0.85 V	0.33 V	0.71 V	143.0	5
CNT@SAC-Co/NCP	0.87 V	0.38 V	0.74 V	172.0	6
$NiCo_2O_4$	0.76 V	0.31 V	0.78 V	71.0	7
FeMn-DSAC	0.92 V	0.40 V	0.71 V	184.0	8
Pt/C +RuO_2	0.84 V	0.31 V	0.70 V	122.0	Our
CoNC-950	0.85 V	0.32 V	0.70 V	181.0	work

References in Supplementary Information

1. E. Y. Choi, D. E. Kim, S. Y. Lee, C. B. Park, C. K. Kim, *Appl. Catal. B-Environ.* **2023**, *325*, 122386.

2. Y. Wang, J. Wu, S. Tang, J. Yang, C. Ye, J. Chen, Y. Lei, D. Wang, *Angew. Chem. Int. Ed.* **2023**, *62*, e202219191.

3. W. Shi, Z. Li, Z. Gong, Z. Liang, H. Liu, Y.-C. Han, H. Niu, B. Song, X. Chi, J. Zhou, H. Wang, B. Y. Xia, Y. Yao, Z.-Q. Tian, *Nat. Commun.* **2023**, *14*, 2294.

4. S.-Y. Lin, Y.-P. Chen, Y. Cao, L. Zhang, J.-J. Feng, A.-J. Wang, *J. Power Sources* **2022**, *521*, 230926.

5. W. Zhang, C. H. Xu, H. Zheng, R. Li, K. Zhou, *Adv. Funct. Mater.* **2022**, *32*, 2200763.

6. J. C. Li, Y. Meng, L. Zhang, G. Li, Z. Shi, P. X. Hou, C. Liu, H. M. Cheng, M. Shao, *Adv. Funct. Mater.* **2021**, *31*, 2103360.

7. H. Li, J. Wang, T. Tjardts, I. Barg, H. Qiu, M. Müller, J. Krahmer, S. Askari, S. Veziroglu, C. Aktas, L. Kienle, J. Benedikt, *Small* **2024**, *20*, 2310660.

8. T. Cui, Y. P. Wang, T. Ye, J. Wu, Z. Chen, J. Li, Y. Lei, D. Wang, Y. Li, *Angew. Chem. Int. Ed.* **2022**, *61*, e202115219.
